# Characterization of the metastatic phenotype of a panel of established osteosarcoma cells

**DOI:** 10.18632/oncotarget.5177

**Published:** 2015-08-13

**Authors:** Ling Ren, Arnulfo Mendoza, Jack Zhu, Joseph W. Briggs, Charles Halsey, Ellen S. Hong, Sandra S. Burkett, James J. Morrow, Michael M. Lizardo, Tanasa Osborne, Samuel Q. Li, Hue H. Luu, Paul Meltzer, Chand Khanna

**Affiliations:** ^1^ Molecular Oncology Section - Metastasis Biology Group, Pediatric Oncology Branch, National Cancer Institute, Bethesda, Maryland, USA; ^2^ Genetic Branch, National Cancer Institute, Bethesda, Maryland, USA; ^3^ Molecular Pathology Unit, Laboratory of Cancer Biology and Genetics, National Cancer Institute, Bethesda, Maryland, USA; ^4^ Comparative Molecular Cytogenetics Core Facility, Center for Cancer Research, National Cancer Institute, Bethesda, Maryland, USA; ^5^ School of Medicine, Case Western Reserve University, Cleveland, Ohio, USA; ^6^ National Institute of Environmental Health, Research Triangle Park, North Carolina, USA; ^7^ Department of Orthopedic Surgery & Rehabilitation Medicine, University of Chicago, Medicine & Biological Sciences, Chicago, USA

**Keywords:** osteosarcoma, PHLDA1/TDAG51, tumor metastasis

## Abstract

Osteosarcoma (OS) is the most common bone tumor in pediatric patients. Metastasis is a major cause of mortality and morbidity. The rarity of this disease coupled with the challenges of drug development for metastatic cancers have slowed the delivery of improvements in long-term outcomes for these patients. In this study, we collected 18 OS cell lines, confirmed their expression of bone markers and complex karyotypes, and characterized their *in vivo* tumorgenicity and metastatic potential. Since prior reports included conflicting descriptions of the metastatic and *in vivo* phenotypes of these models, there was a need for a comparative assessment of metastatic phenotypes using identical procedures in the hands of a single investigative group. We expect that this single characterization will accelerate the study of this metastatic cancer. Using these models we evaluated the expression of six previously reported metastasis-related OS genes. Ezrin was the only gene consistently differentially expressed in all the pairs of high/low metatstatic OS cells. We then used a subtractive gene expression approach of the high and low human metastatic cells to identify novel genes that may be involved in OS metastasis. PHLDA1 (pleckstrin homology-like domain, family A) was identified as one of the genes more highly expressed in the high metastatic compared to low metastatic cells. Knocking down PHLDA1 with siRNA or shRNA resulted in down regulation of the activities of MAPKs (ERK1/2), c-Jun N-terminal kinases (JNK), and p38 mitogen-activated protein kinases (MAPKs). Reducing the expression of PHLDA1 also delayed OS metastasis progression in mouse xenograft models.

## INTRODUCTION

Osteosarcoma is the most common bone sarcoma in pediatric patients. [1, 2]. The etiology and molecular mechanisms of OS remain unclear, although alterations of *Rb*, *p53, mdm2*, and *myc* have been identified in many cases [1, 3–6]. Pulmonary metastasis occurs early in the natural history of OS and is a major cause of mortality and morbidity [2]. Most patients have subclinical micrometastasis at initial diagnosis. Survival has remained unchanged for the past 30 years [7]. Despite complete and definitive surgical resection of the primary lesion, nearly 90% of OS patients develop metastasis. Further, 10-20% of patients have clinically detectable lung metastasis at presentation and 80% of those patients will relapse. Long-term (5 years) survival rate for localized OS is less than 75%.

Limited improvement in outcomes and survival of OS patients has been seen since the introduction of chemotherapy in the 1970's [8–10]. For such rare tumors, with chaotic genetics, lack of recurrent genetic alterations, early onset, and aggressive behavior, there is need for a panel of preclinical models that can capture some of the genetic heterogeneity and more importantly will allow the study of metastasis. A variety of OS cell lines have been described *in vitro*; however significant differences in the *in vivo* phenotypes have been reported and often limit the opportunity to make assessments across studies. In this study, we, for the first time in a single research group, evaluated the *in vitro* and *in vivo* phenotypes of 18 frequently used OS cell lines. We compared and characterized each cell line for their tumorgenicity and the metastatic potential in transplantable murine models. Furthermore a priority was placed on the evaluation of metastatic potential in these cells. Four murine OS cell lines (K12/K7M2, DUNN/DLM8) and fouteen human OS cell lines (TE85/HOS/HOS-MNNG/143B/Krib, MG63/MG63.2/MG63.3, SaOS/LM7, Hu09/Hu09-M112/Hu09-M132/Hu09-H3) with low/high metastatic phenotypes were identified and selected for the *in vivo* metastasis experiments. We validated the identity of the selected high/low metastatic human OS cell lines by STR (Short Tandem Repeat) DNA profiling. The expression of bone differentiation markers in each parental OS cell line was evaluated by RT-PCR, and the characteristic complexity of their genomes, assessed by karyotype analysis. Global mRNA expression analysis was then performed using these cell lines to identify potential metastasis related genes in OS.

From the expression subtraction of high and low metastatic cells, PHLDA1 (also known as T-Cell Death-Associated Gene 51 (TDAG51)) was identified as one of the genes with higher expression in highly metastatic OS cells. PHLDA1 was first described to have a role in the induction of the death receptor CD95/Fas gene expression and activation-induced-apoptotic cell death (AICD) in response to the engagement of the T-cell receptor in a murine T-cell hybridoma [11]. However, to date, several reports demonstrate that TDAG51 may have both pro- and anti-apoptotic functions depending on the cellular context and circumstances. The reports which related to the pro-apoptotic function of TDAG51 support the notion that the expression of TDAG51 is highly induced by homocysteine and heat shock stress and it promotes apoptotic cell death [12]. In addition, the process of tumorigenesis is enhanced by the down regulation of TDAG51, which results in the inhibition of tumor cell apoptosis [13]. However, in contrast to its pro-apoptotic function, TDAG51 has also been shown to have an important role in insulin-like growth factor-1-induced cell survival [14]. TDAG51 can also prevent the oxidative stress induced cell death [15]. In addition, several groups reported that TDAG51 expression is highly enhanced in some tumor types such as colon and intestinal tumors, indicating that TDAG51 may be involved in tumor cell proliferation but not in apoptotic cell death [16, 17]. In our study, using the OS cell lines with differential metastatic potential, we identified PHLDA1 (TDAG51) was expressed at a higher level in the high metastatic OS cells comparing to low metastatic OS cells. Subsequently, knock down of TDAG51 expression using siRNA or shRNA resulted in reduced metastasis *in vivo* and also reduced the activities of MAPKs (the extracellular-signal-regulated kinases 1/2 (ERK1/2), c-Jun N-terminal kinases (JNK), and p38 mitogen-activated protein kinases (MAPKs). However the mechanisms whereby TDAG51 inhibits metastasis are unclear.

## RESULTS

### Collection and *in vitro* characterization of OS cell lines

As shown in Table 1, we collected 18 established OS cell lines which included 4 murine and 14 human OS cell lines. All the cell lines were tested for mycoplasma contamination and all were negative except MG63.2. Therefore we developed a mycoplasma-free variant, MG63.3 cell line, using *in vivo* passage, as described in separate work by our group [18].

**Table 1:** Summary of primary tumor growth, spontaneous and experimental metastasis behaviors of OS cell lines

Osteosarcoma cell lines	Species	% Tumor take in mice	Days of primary tumor reach 1.5-2 cm3	% Generation of spontaneous metastasis	Median survival days of spontaneous metastasis	% Generation of experimental metastasis	Median survival days of experimental metastasis	References
TE85	Human	80%	Not achieved at day 200	0%	Not achieved at day 200	0%	Not achieved at day 120	[50]
HOS	Human	90%	Not achieved at day 200	0%	Not achieved at day 200	0%	Not achieved at day 120	[50]
HOS-MNNG	Human	100%	32	100%	65	100%	29	[51]
143B	Human	100%	27	100%	44*	100%	29	[52]
Krib	Human	100%	37	100%	59	100%	36	[53]
SaOS	Human	100%	95	100%	154*	100%	Not achieved at day 120	[54]
LM7	Human	100%	91	100%	123	100%	65	[54]
MG63	Human	20%	170	0%	Not achieved at day 200	0%	Not achieved at day 120	[55]
MG63.2	Human	100%	45	100%	96	100%	25	[56]
MG63.3	Human	100%	40	90%	83	100%	25	
Hu09	Human	100%	180	100%	Not achieved at day 200	90%	Not achieved at day 120	[57]
Hu09-M112	Human	100%	89	100%	194	100%	97	[58]
Hu09-M132	Human	100%	78	100%	154	80%	84	[58]
Hu09-H3	Human	100%	67	100%	133	100%	60	[59]
DUNN	Mouse	100%	25	100%	74*	50%	Not achieved at day 120	[60]
DLM8	Mouse	100%	25	100%	40*	80%	24*	[60]
K12	Mouse	40%	170	0%	Not achieved at day 200	20%	Not achieved at day 120	[61]
K7M2	Mouse	100%	28	80%	100	100%	29	[61]

* Metastasis occurs in other organs besides lung

The origins of the OS cell lines are as shown in Table 1 with publication references. Cytogenetic karyotyping was performed for each OS cell line which confirmed their species of origin and complex genetic landscapes. All the cells showed a highly degree of aneuploidy, a characteristic hallmark of OS (data not shown). Bone differentiation genes (osteocalcin, alkaline phosphatase, osteopontin, osteomodulin, osterix) of parental human OS cell lines (HOS, Hu09, SaOS and MG63) were evaluated by RT-PCR and as shown in Supplementary Table 2. The RT-PCR results were presented as detectable or undetectable bands. Hu09 was the only cell line expressing all bone related genes that we evaluated. [19]. To further confirm their origin, the mesenchymal cell marker protein-vimentin was detected by immunohistochemistry staining and Western blot analysis for all the OS cell lines (data not shown).

### *In vivo* characterization of OS cell lines

**Figure 1:**
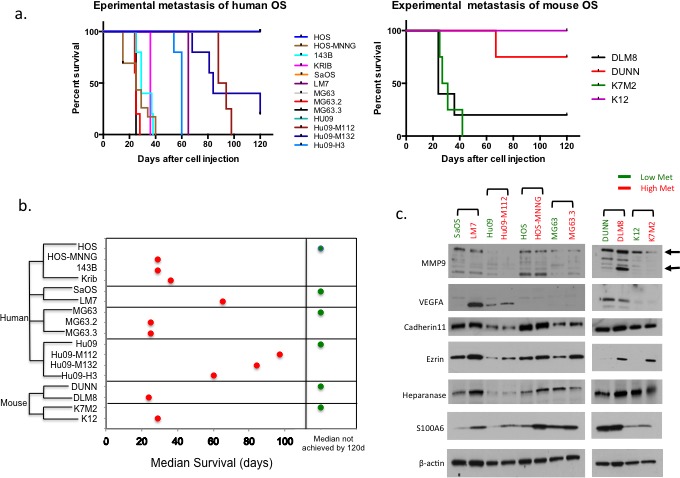
The metastatic potentials of OS cell lines and the expression of metastatic related genes in high/low metastatic OS cell lines **a.** Kaplan-Meier Survival curves of human and mouse OS cell lines in experimental metastasis experiments. **b.** Summary of the median survival days in the clonally related OS cell lines. **c.** Western blot analysis of the expression of previously reported metastasis related genes in the pairs of high/low metastatic OS cell lines.

The *in vivo* tumor growth and metastasis of murine OS cell line K7M2 and K12 were tested in Balb/c mice, DUNN and DLM8 in C3H mice. All the human OS cell lines were tested in SCID mice using previously described experimental metastasis and spontaneous metastasis assays (Materials & Methods) [18]. The primary tumor growth rate, metastatic potential and median survival of each cell line evaluated in both experimental metastasis and spontaneous metastasis models are shown in Table 1. For all the OS cell lines we tested using the spontaneous metastasis model, TE85, HOS, Hu09, MG63 and K12 all had much slower primary tumor growth rates and had no or very low incidence of pulmonary metastasis at the experiment termination time (200 days). HOS-MNNG, MG63.2, MG63.3, 143B, Krib, Hu09-M112, Hu09-M132, Hu09-H3, LM7, SaOS, K7M2, DLM8 and DUNN yielded orthotopic primary tumors and then spontaneous metastasis to the lung (Supplementary Figure 1a &1b). When testing the cell lines in the experimental metastasis model, the median survival days of the pulmonary metastasis of all the parental cell lines (MG63, HOS, Hu09, SaOS, K12 and DUNN) were greater than 120 days, while the median survival days of highly metastatic cell lines (MG63.2, MG63.3 and DLM8) were as short as 25 days (Table 1, Figure 1a & 1b).

Most OS cells metastasize to the lung, but SaOS, 143B, DUNN and DLM8 cells also metastasized to other distant organs including lymph nodes, liver, adrenal gland, kidney or ovary. Following *in vivo* characterization, the histology of all resulting tumors was evaluated by the veterinary pathologist and found to be consistent with sarcoma with varying degrees of osteoid present (data not shown). The highly metastatic OS cell lines tested in this study were derived from their low metastatic parental cells by chemical treatment (HOS-MNNG), or through metastatic selection in the mouse (LM7, MG63.2, MG63.3, K7M2, Hu09-M112, Hu09-M132, DLM8), or single clone selection in cell culture (Hu09-H3).

Among the OS cell lines, there were 6 clonally related groups of cell lines that differed based on metastatic potential, (K12/K7M2, DUNN/DLM8; murine) and (HOS/HOS-MNNG/143B/Krib, MG63/MG63.2/MG63.3, SaOS/LM7, Hu09/Hu09-M112/Hu09-M132/Hu09-H3; human). In each group of cell lines, the highly metastatic line(s) is clonally related to the low metastatic line (Figure 1b). The authentication of human cell lines was further confirmed with STR DNA profiling by the University of Colorado DNA Sequencing & Analyses Core. The highly metastatic cell lines fully match their parental cell lines (data not shown).

Following this rigorous *in vitro* and *in vivo* characterization, we then asked if the high/low metastatic pairs could be distinguished based on the expression of previously reported metastasis associated genes. The expression of previously reported metastasis associated genes, MMP9 [20], Ezrin [21], VEGFA [22], Cadherin11 [23], S100A6 [24], and Heparanase [25] were evaluated in these high/low OS cell lines. As shown in Figure 1c, Ezrin was the only gene consistently shown differentially expressed in all the pairs of high/low metastatic OS cells.

### Gene expression analysis

**Figure 2:**
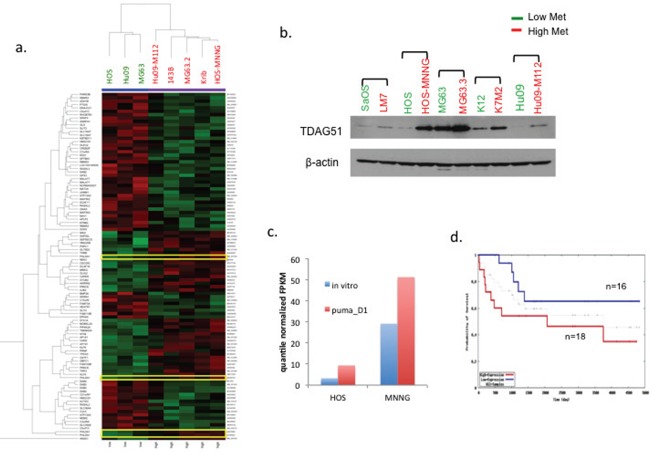
High PHLDA1/TDAG51 expression is associated with high metastatic potential in OS cell lines and lower survival probability in OS patients **a.** Expression array heat map shows the genes selected by *p* < 0.01 and >2 fold change between high and low metastatic OS cells. Four probes for the PHLDA1/TDAG51 gene are highlighted. **b.** Western blot analysis shows the protein expression of TDAG51 in pairs of high/low metastasis OS cell lines. Green label: low met cells: red label: high met cells. **c.** Comparison of PHLDA1/TDAG51 expression with RNA Seq on HOS and MNNG cells *in vitro, as well as* one day in PuMA. **d.** Kaplan-Meier Survival estimation shows pediatric OS patients with high TDAG51 expression (red line) (*n* = 18) had lower survival probability than the patients with low TDAG51 (blue line) (*n* = 16).

Three human OS cell lines (HOS, MG63 and Hu09) were compared to their highly metastatic derivatives (HOS-MNNG, 143B, Krib, MG63.2 and Hu09-M112) for differential gene expression by DNA microarray analysis to identify commonly regulated genes that might contribute to tumor progression and metastasis. Figure 2a shows the genes selected by *p* < 0.01 and > 2 fold change between high and low metastatic cells. PHLDA1 was one of the genes with increased expression in highly metastatic cell lines. Notably, all four probes for PHLDA1 gene were consistently differentially expressed between high and low metastatic OS cells. We then expanded our analysis to the protein expression of TDAG51 (PHLDA1 gene product) in more pairs of high/low metastatic OS cell lines. As shown in Figure 2b, TDAG51 protein level was also higher in the highly metastatic OS cells compared to their poorly metastatic parental cells in 5 pairs of OS cell lines. The differential gene expression of TDAG51 was also confirmed with RNA-seq analysis in two high/low metastatic OS cell lines (HOS/HOS-MNNG, MG63/MG63.3) (Figure 2c). In addition to the *in vitro* data, we also observed that TDAG51 expression was increased when the OS cells resided in the lung (PuMA day1) (Figure 2c, Supplementary Figure 2). We then used an existing OS patient database [26] to ask if the expression of PHLDA1/TDAG51 correlated with the outcomes of OS patients. As shown in Figure 2d, the OS patients with lower expression of TDAG51 had a higher tendency to survive longer.

### Reduction of OS cells pulmonary metastasis by TDAG51 knock down

**Figure 3:**
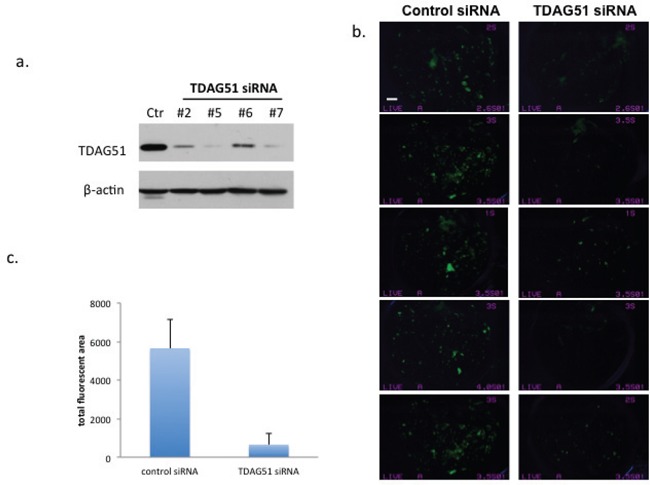
Suppression of TDAG51 expression reduced pulmonary metastasis of OS cells **a.** Western blot analysis of TDAG51 knockdown in MG63.3/GFP cells with 4 siRNA probes. **b.** Assessment of early metastatic progression of TDAG51 knockdown MG63.3/GFP cells (siRNA #5 was used for knockdown). Fluorescent microscope images of lungs (*n* = 5 in each group) 17 days post injection of OS cells, in which green fluorescent events represent tumor cells (bar, 2 mm). **c.** Quantification of total fluorescent green tumor cells in the lungs. There is statistically significant reduction in tumor cells following TDAG51 knock down compared to the control group (*p* = 0.0001).

To study the relationship of TDAG51 with OS tumor metastasis, we performed gene knock down with TDAG51 siRNA in the highly metastatic MG63.3/GFP and HOS-MNNG/GFP cells. The TDAG51 knock down was evaluated by Western blot analysis using different siRNA sequences (Figure 3a), and the reduction of TDAG51 expression did not change the cellular morphology, viability and proliferation rate of MG63.3/GFP cells (data not shown). MG63.3/GFP control cells and TDAG51 knock down cells (10^5^ cells/mouse) were intravenously injected into SCID mice (*N* = 5 per group). Seventeen days after injection, the mice were euthanized and the lungs were extracted and imaged with a fluorescent dissecting microscope. The total fluorescent area in each lung was calculated. As shown in Figures 3b & 3c, there is a statistically significant reduction in tumor cells following TDAG51 knock down compared to the control group (*p* = 0.0001). To confirm these results in a second model, this *in vivo* experiment was repeated using HOS-MNNG/GFP cells. Again, the lung micro-metastases were quantified using inverted fluorescence microscopy (Supplementary Figure 3a).

**Figure 4:**
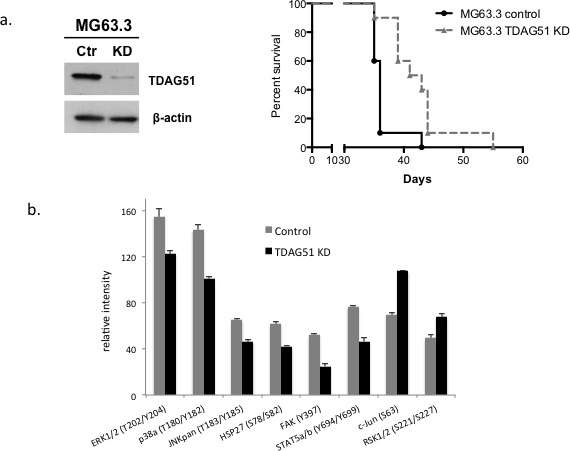
Suppression of TDAG51 expression improved overall survival in a mouse model of OS pulmonary metastasis and reduced the kinase activities of MAPK signaling pathway members **a.** Western blot analysis of TDAG51 knockdown in MG63.3/GFP cells with shRNA. **b.** Experimental metastasis experiments were performed by tail vein injection of highly metastatic OS cells (MG63.3/GFP) transfected with TDAG51 siRNA or control siRNA. Tumor bearing mice with TDAG51 shRNA expressing cells had a significantly longer survival rate compared to control mice (*p* = 0.026). **c.** Kinase array assay using cell lysates from MG63.3/GFP cells stably expressing TDAG51 shRNA or control shRNA. Knock down of TDAG51 expression significantly reduced phosphorylation of kinases in the MAP kinase signal transduction pathway.

To further confirm the inhibition of metastasis following knock down of TDAG51 expression in OS cells, experimental metastasis experiments were performed by tail vein injection of highly metastatic OS cells (MG63.3/GFP) stably expressing TDAG51 shRNA or control shRNA. As expected, mice injected with TDAG51 shRNA expressing cells had a significantly higher survival rate compared with control mice (*p* = 0.026) (Figure 4a). The same experiment was repeated with HOS-MNNG/GFP cells and a similar result was observed (Supplementary Figure 3b).

To understand the mechanisms associated with TDAG51 and its effect on metastasis, we evaluated cell-signaling pathways that were altered by knocking down TDAG51 expression in highly metastatic OS cells. Kinase array assays were performed using cell lysates from MG63.3/GFP cells stably expressing TDAG51 shRNA or control shRNA. As shown in Figure 4b, knock down of TDAG51 expression significantly reduced phosphorylation of kinases in the MAP kinase signal transduction pathway such as ERK1/2, P38a and JNK.

## DISCUSSION

Osteosarcoma is the second highest cause of cancer-related death in the pediatric age group and the most common primary tumor of the bone. Improvements in the survival rates have not changed substantially in over 30 years [27, 28]. This is partly due to a lack of good understanding of the biology of this complex cancer, its rareness and the limited understanding of its metastasis biology. To overcome this problem, the establishment and use of well-characterized osteosarcoma models and the development of novel models is essential. Tumor-derived cell lines could be used in *in vitro* and *in vivo* models if they are representative of the human condition and if they allow the study of metastasis [29–31].

Although numerous OS cell lines have been established *in vitro*, there are few reliable and reproducible *in vivo* descriptions available. Since pulmonary metastasis is the main cause of morbidity in OS, developing and establishing OS cell lines and animal models, which can model the spectrum of human metastatic OS, represents a valuable tool in the ongoing study of this devastating disease. In this report, we collected a large panel of osteosarcoma cell lines. We then for the first time rigorously evaluated their *in vivo* behavior employing standardized methods and animal procedures within a single laboratory group. All the OS cell lines we tested produced tumors orthotopically and in 15 cases developed pulmonary metastases. These cell lines were verified by karyotype analysis for their respective species of origin and identified to be highly aneuploid, as expected in osteosarcoma. Further, the resulting tumors were consistent with osteosarcoma with variable degrees of bone differentiation. The tumorigenic and metastatic OS cell lines selected in this study are believed to provide a valuable panel of reagents to capture the heterogeneity of this disease and to study metastases in the disease. Accordingly, we believe that the OS models used in this study provide potential value to pediatric osteosarcoma researchers, and expect that our rigorous characterization and expression analysis to fuel ongoing studies in the field.

From the 18 OS cell lines evaluated in this study, we selected 6 groups of high and low metastatic cell lines for the further studies on OS metastasis *in vitro* and *in vivo*. In each group, the highly metastatic cell lines are clonally related to their low metastatic counterparts. As previously reported [31, 32], the 143B cell line, derived from HOS by transfection with the k-ras oncogene, showed aggressive progression and highly metastatic phenotype in our study. But 143B cells metastasized to various organs including lung, ovary, kidney, adrenal gland, and liver. HOS-MNNG cells has been reported as non metastatic in other studies [31, 33], but we found they consistently metastasized to the lung in both the experimental and the spontaneous metastasis mouse models in our studies. In this study, we also observed relative aggressiveness of the low metastatic parental cell lines (SaOS and Hu09) comparing to the previous reports. All of these differences may be accounted for by the discrete batches of cell lines, distinct methods of injection and distinct mouse strains (Nude vs. SCID) [34].

The clonally related high/low metastatic OS cell lines provide excellent models to study OS metastasis. First, the highly metastatic cell lines are all generated from their low metastatic parental cell lines and therefore, should have close genetic backgrounds. Second, all the metastatic cell lines produced lung metastases within 120 days when they were injected through the tail vein and finished the spontaneous tumor metastasis cycle in 200 days, which are the very important features for a good model to study tumor metastasis. Third, each pair of high/low metastatic OS cell lines have different genetic backgrounds and were generated from tumors of different osteoblast differentiation stages and therefore could represent a wider range of OS patients conditions. This may ultimately help us to better understand the extreme genetic variability of this disease, while providing the necessary tools to comparatively study OS progression and metastasis, which is the major cause of morbidity and mortality in patients.

The analysis of differentially expressed genes by microarray, comparing metastatic OS cell lines to parental low metastatic cell lines, should help to identify common pathways or even a set of proteins that regulate OS tumor progression and metastasis. We and others have previously used such approaches to identify metastasis-associated differences in gene expression, within small subsets or pairs of high and low metastatic potential cells, but it has not previously been possible to include a well characterized panel of high and low metastatic cells in this type of analysis. In this study, we identified PHLDA1 (TDAG51), one of the genes up-regulated in all the highly metastatic OS cell lines compared to their low metastatic parental cells. The RNA-seq study also demonstrated the differential expression of TDAG51 between high/low metastatic OS cells at the early stage of lung metastasis. The survival estimation of OS patients with high/low expression of TDAG51 showed an association between high TDAG51 expression and poor outcome in pediatric osteosarcoma patients. However, due to the limited number of OS patient sample data available for this particular analysis, the results were not statistically significant. Recent publications showed that TDAG51 (PHLDA1) is also highly expressed in other tumor types such as colon and intestinal tumors, which represents epithelial stem cell features [16, 17]. In addition, it has been reported that TDAG51 (PHLDA1) expression is induced by external stress such as heat shock, and modulated by IGF-I and ERK pathways [12, 14, 35, 36]. In a study of gene expression on high/low metastatic OS cell lines, TDAG51 (PHLDA1) was reported to be up-regulated in two highly metastatic OS cell lines (143B, K7M2), but down regulated in another two high metastatic OS cell lines (MG63-M8, SaOS/LM5) [37]. However, in this previous study, the authors mistakenly identify TSSC3 as an alias for the PHLDA1 gene. Rather, TSSC3 is the alias for the related PHLDA2 gene, not PHLDA1, and therefore it is difficult to interpret these previous findings [38].

In our gene knock down study, reducing expression of TDAG51 in highly metastatic human OS cells (MG63.3) resulted in a reduction of phospho-ERK1/2 and phospho-p38 and inhibition of their lung metastasis *in vivo*.

It has been reported that inhibition of ERK1/2 leads to increased apoptosis and decreased metastasis in OS studies [39, 40]. The inhibition of phospho-ERK1/2 resulted in the inhibition of OS cell motility *in vivo* and slower tumor growth and prolonged survival by inducing the production of pro-apoptotic proteins [41]. MAPK pathway also plays a major role in the early survival of Ezrin dependent metastatic OS cells in the lung [21]. More intensive studies are needed to reveal the functional role of TDAG51 in OS cell metastasis.

For the past 30 years, the studies of OS have widened our knowledge about this aggressive malignancy. The high genetic instability of the primary tumor, the rareness of the disease and poor access to primary patient material due to intensive neoadjuvant treatment regimens hamper biological studies. Therefore, multiple representative models are needed to gain more insight into different processes involving OS initiation, progression and treatment. The pairs of high/low metastatic OS cell lines which we characterized in this study will provide very useful models for studying the molecular mechanism of OS metastasis and for testing or screening for novel therapeutic agents to treat the disease.

## MATERIALS AND METHODS

### Cells

Human OS cell lines TE85, HOS. SaOS, Krib, 143B, MG63, HOS/MNNG were purchased from the American Type Culture Collection (ATCC). Hu09, Hu09-H3, Hu09-M132 and Hu09-M112 were obtained from Dr. Jun Yokota (National Cancer Center Research Institute, Tokyo, Japan). SaOS-LM7 was from Dr. Eugenie S. Kleinerman (The University of Texas: MD Anderson Cancer Center, Houston, TX). MG63.2 cells were from Dr. Hue Luu (University of Chicago Medicine, Chicago, IL). MG63.3 cells were derived from MG63.2 by a process of experimental metastasis [18].

Murine OS cell lines DUNN and DLM8 were from Dr. Hideki Yoshikawa (Osaka medical center for cancer and cardiovascular diseases, Osaka, Japan). K12 and K7M2 were developed in our lab [18].

All the cells were cultured in DMEM (Invitrogen, Carlsbad, CA) medium supplement with 10% fetal bovine serum (FBS) and glutamine except Hu09 series that are cultured in RPMI 1460 with 10% FBS and glutamine.

Mycoplasma test were routinely performed with Mycoalert Mycoplasma detection kit (Lonza Group Ltd, Basel, Switzerland).

### *In vivo* tumor formation and metastasis

After trypsinization, cells were counted and dilutions of 2×10^6^ viable cells in 100 μl HBSS were injected orthotopically to the para-osseous proximal tibia as previously described [18]. The tumors were measured every 3-4 days. The volume of orthotopic tumor was calculated as previously reported [42]. Tumor bearing limbs were resected at a tumor size of 1.5-2.0 cm^3^. Mice were then evaluated every other day for the development of metastasis-associated morbidity using pulmonary assessment of advanced metastasis (PAAM) method [43]. The experiments were terminated if there was no sign of tumor formation or metastasis related death 200 days after tumor cell injection.

For experimental metastasis assays, 10^6^ OS cells in 100 μl HBSS were intravenously injected into the mice. The experiments were terminated if there was no metastasis related death at 120 days.

For both experimental and spontaneous metastasis assays, 5 mice were used for the primary screen and 10 mice were included for the replicated experiments. BALB/c mice were used for K7M2 and K12 OS cells, C3H mice were used for DUNN and DLM8 and SCID mice were used for all the human cell lines. Complete necropsy allowed the identification and validation of pulmonary metastases in all mice. All primary tumors and the whole lungs with the metastases were archived by fixing in formalin and embedding in paraffin. All animal studies were done with the approval of the Animal Care and Use Committee of the National Cancer Institute.

### Western blot

Western blot analysis was performed as described previously [44]. Cultured cells were lysed in SDS gel loading buffer or RIPA buffer with protease inhibitor cocktail (Roche Life Science, Indianapolis, IN) and phosphate inhibitor cocktail (Thermo Scientific, Rockford, IL), equal amount of proteins were loaded on 4-12% or 4-20% SDS-PAGE gels (Invitrogen). After electrophoresis, proteins were transferred to a nitrocellulose membrane and probed with primary antibodies of Ezrin, β-Actin (Sigma, St. Louis, MO), TDAG51 (Santa Cruz Biotechnology, Inc. Dallas, Texas), VEGFA (Proteintech Group, Inc., Chicago, IL), VEGFR2 (Cell Signaling Technology, Inc. Boston, MA), Cadherin11 (Invitrogen), Heparanase1, Vimentin (Abcam, Cambridge, MA), S100A6 (Gift from Dr. Mark Simpson's lab, NCI/NIH), MMP9 (Proteintech).

### DNA array and data analysis

Total RNA samples from all the OS cells were prepared using Qiagen RNA mini kit (Qiagen, Hilden, Germany) according to manufacturer's directions. RNA quality was assessed using an Agilent 2100 Bioanalyzer. All samples were prepared for cRNA hybridization via the Affymetrix One-cycle Eukaryotic Target Labeling Assay according to manufacturer's instructions. Once the cRNA was cleaned and fragmented, it was individually hybridized to Affymetrix either Mouse Genome 430 2.0 arrays or Human Genome U133 Plus 2.0 arrays. All samples were prepared and hybridized at the National Cancer Institute (NCI) DNA array core facility.

The .CEL files were exported from Affymetrix GCOS software and further data process and analysis was done in R/Bioconductor software (http://www.bioconductor.org). Briefly, data normalization was performed with Robust Multi-array Average (RMA) procedure; differentially expressed analysis was done by the affy and simpleaffy packages. Adjustment of P-values was done by Benjamini and Hochberg method [45]. The top differentially expressed genes were selected by adjusted P-values and log fold change. The dendrogram was generated with distance calculated by Euclidean method and hierarchical clustering was done using method of “average”.

### RNA Sequencing and Data Analysis

Gene expression profiles of cell lines grown *in vitro* were compared to expression profiles of the same cell lines at early time points during metastatic colonization using the *ex vivo* pulmonary metastasis assay (PuMA) [46]. Briefly, 1×10^6^ GFP+ cells (HOS/HOS-MNNG, MG63/MG63.3) were injected into the tail veins of SCID mice. The mice were immediately sacrificed and their lungs were insufflated with a media/agarose mixture, transversely sectioned, and grown in *ex vivo* culture on gel foam at the media air/fluid interface. Twenty-four hours after injection, the lungs were mechanically homogenized and GFP+ tumor cells were separated from mouse lung cells by FACS. RNA was extracted with TRIzol (Invitrogen) and purified using the RNeasy Plus kit (Qiagen). *In Vitro* cultured cells were trypsinized, subjected to mechanical homogenization and the same downstream processing as cells isolated from PuMA. RNA quality was assessed using an Agilent 2100 Bioanalyzer. PolyA+ RNA was prepared for sequencing using the Illumina TruSeq RNA Sample Preparation Kit according to the manufacturer's protocol. RNA-seq libraries were sequenced on the Illumina HiSeq 2000 platform at the Case Western Reserve University Next Generation Sequencing Core Facility.

For gene expression analysis, reads were aligned to the hg19 genome build (retrieved from http://cufflinks.cbcb.umd.edu/igenomes.html), using Tophat v2.0.7 [47]. FPKM values for known genes were calculated using Cufflinks v2.0.2 [48] provided with the GTF file via the -G (known genes only) option. FPKM values were tabled by converting background values (<0.3) to 0 and adding 0.3 to all values [49]. FPKMs were quantile normalized across all samples.

### TDAG51 knock down with short interfering RNA (siRNA) or short hairpin RNA (shRNA)

MG63.3/GFP or HOS-MNNG/GFP (10^5^ cells/well) cells were seeded in the 6-well plate one day before transfection. The cells were transfected with a mix of 5 μl of Lipofectamine RNAiMAX Reagent (Invitrogen) and 30 pmole of individual human PHLDA1 (TDAG51) siRNA or a pool of 4 siRNAs (Qiagen). The knock down efficiency was evaluated by western blotting.

For the knock down of TDAG51 with shRNA, MG63.3/GFP or HOS-MNNG/GFP cells were seeded in the 6-well plate (10^5^ cells/well) one day before transfection. The cells were infected with lentiviral suspensions (10^6^TU/well or MOT=5) containing non-target control vector or containing pLKO.1-shTDAG51 (Sigma). The stably transfected cells were selected and maintained with puromycin. The knock down efficiency was evaluated by Western blot analysis.

### Early pulmonary metastasis assessment

MG63.3/GFP cells were transfected with control siRNA and TDAG51 siRNA for 24 hours, then harvested and intravenously injected (10^5^ cells in 100 μl HBSS) into SCID mice. The mice were sacrificed at 17 days after injection. The lungs were extracted and imaged using Leica MZFLIII dissecting fluorescent microscope. The total areas of GFP fluorescence were calculated with Openlab software (Improvision Ltd., Coventry, England).

This experiment was also repeated in HOS/MNNG/GFP cells. 5×10^5^ cells were injected into female SCID mice. The green fluorescent micro lung metastases were counted using a Leica DMIRB inverted fluorescent microscope.

### Human Phospho-kinase array

TDAG51 siRNA and scrambled siRNA were transfected into MG63.3 cells. Forty-eight hours later, cell lysates were collected from 10^7^ TDAG51 knock down cells and control cells. Total protein of 200 μg was used for the immunoblotting with human phospho-kinase array. The experiment was performed following the manufacture's instruction (B&D systems, Minneapolis, MN). Image J was used to quantify the intensity of the Western blot signals.

## SUPPLEMENTARY MATERIAL FIGURES AND TABLES


