# Supplementary material for: Characterization of the metastatic phenotype of a panel of established osteosarcoma cells
Source: Oncotarget. 2015 Aug 13;6(30):29469–81. doi: 10.18632/oncotarget.5177 (PMC4745740; doi:10.18632/oncotarget.5177)
Supplement: Supplementary file 1 [file oncotarget-06-29469-s001.pdf]

Characterization of the metastatic phenotype of a panel of established osteosarcoma cells

Supplementary Material

Supplementary figure 1

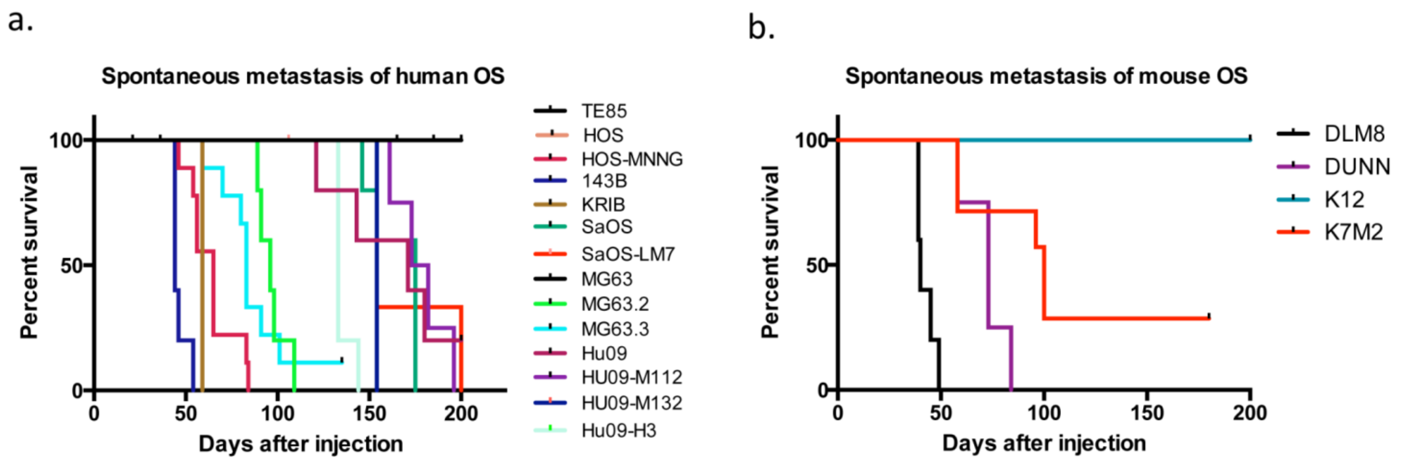

Kaplan-Meier Survival curves of 13human OS cell lines (a) and 4 murine OS cell lines (b) in the spontaneous metastasis experiments.

Supplementary figure 2

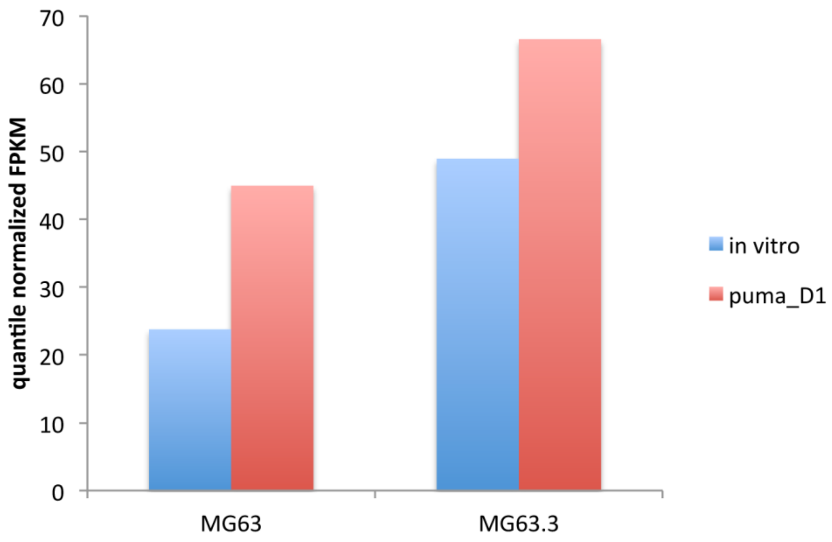

Comparison of PHLDA1/TDAG51 expression with RNA-Seq on MG63 and MG63.3 cells *in vitro*, as well as one day in pulmonary metastasis assay (PuMA).

Supplementary figure 3

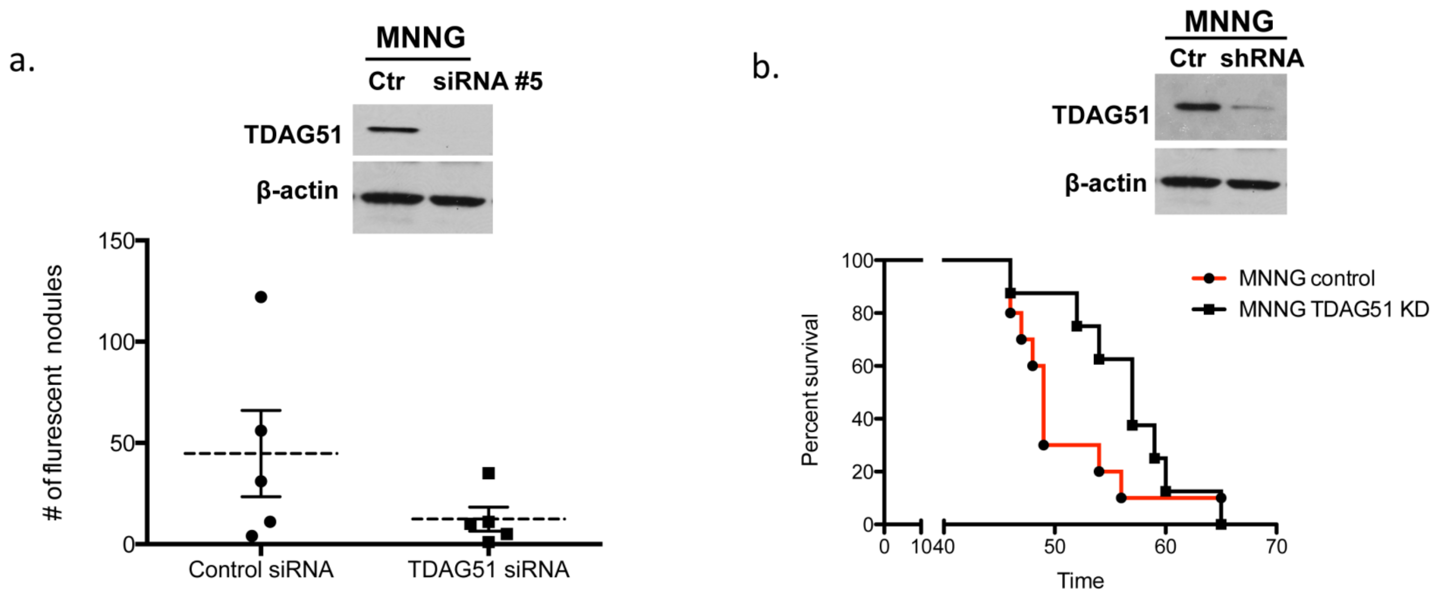

Suppression of TDAG51 in HOS-MNNG (another highly metastatic OS cell line) reduced pulmonary metastasis. (a). Western blot analysis of TDAG51 knockdown in HOS-MNNG/GFP cells with siRNA and the quantification of total green fluorescent tumor nodules in the lungs (n=5 in each group) after 17 days of cancer cell injection. (b). Experimental metastasis experiments were performed by tail vein injection of HOS-MNNG/GFP stably expressing TDAG51 shRNA or control shRNA (insert western blot). Mice bearing TDAG51 shRNA expressing cells had delayed metastasis related death.

Supplementary table 1. Primers for RT-PCR

Protein name	Sense primer	Antisense primer
Alkaline phosphatase	5’-acgtggctaagaatgtcatc-3’	5’-ctggtaggcgatgtcctta-3’
Osteopontin	5’-ccaactaagtccaacgaaag-3’	5’-ggtgatgtcctcgtctgta-3’
Osteocalcin	5’-atgagagccctcacactcctc-3’	5’-gccgtagaagcgccgataggc-3’
Osteomodulin	5’-caaacaggattcccatttcgtca-3’	5’-gttgctgaatgtgcatcggaat-3’
Osterix	5’-actttggatgctcccatctccacct-3’	5’-agggcatgatcccttccattccaca-3’

RT-PCR

RNA was isolated using Trizol® LS reagent according to manufacturer's instructions (Invitrogen). RNA was subjected to an additional purification step using the RNeasy spin column clean up protocol supplied by the manufacturer (Qiagen). Samples were quantitated using a Nanodrop apparatus and 1 µg of total RNA was reverse transcribed. A Universal Mouse Reference RNA (Stratagene, La Jolla, CA) was used as a positive control and negative controls were included as reactions containing no cDNA template. Reverse transcription was primed using random hexamers and cDNA synthesis conducted using Moloney murine leukemia virus reverse transcriptase (Promega Corporation, Madison, WI) at 37°C for 1 hr. The reaction was halted by heating at 70°C for 10 min. Two microliter of the cDNA reaction was used for PCR reaction. Thermocycling parameters were as follows: step 1 = 95°C for 3 min., step 2 = 95°C for 40 sec., step 3 = 61°C for 40 sec., step 4 = 68°C for 40 sec. with steps 2–4 repeated 27 additional times. The PCR products were separated on 1% agarose gel. The primers were used at a final concentration of 100nM for gene specific amplification as shown in supplementary Table 1.

Supplementary table 2. RT-PCR analysis of osteoblast markers expressed in human OS cell lines

	HOS	MG63	SaOS	Hu09
**Alkaline phosphatase**	+	+	+	+
**Osteopontin**	+	-	+	+
**Osterix**	-	-	+	+
**Osteocalcin**	-	+	-	+
**Osteomodulin**	-	-	-	+
